# Inbreeding depression due to recent and ancient inbreeding in Dutch Holstein–Friesian dairy cattle

**DOI:** 10.1186/s12711-019-0497-z

**Published:** 2019-09-27

**Authors:** Harmen P. Doekes, Roel F. Veerkamp, Piter Bijma, Gerben de Jong, Sipke J. Hiemstra, Jack J. Windig

**Affiliations:** 1 Wageningen University & Research, Animal Breeding and Genomics, P.O. Box 338, 6700 AH Wageningen, The Netherlands; 2 Wageningen University & Research, Centre for Genetic Resources the Netherlands, P.O. Box 16, 6700 AA Wageningen, The Netherlands; 3 Cooperation CRV, Wassenaarweg 20, 6843 NW Arnhem, The Netherlands

## Abstract

**Background:**

Inbreeding decreases animal performance (inbreeding depression), but not all inbreeding is expected to be equally harmful. Recent inbreeding is expected to be more harmful than ancient inbreeding, because selection decreases the frequency of deleterious alleles over time. Selection efficiency is increased by inbreeding, a process called purging. Our objective was to investigate effects of recent and ancient inbreeding on yield, fertility and udder health traits in Dutch Holstein–Friesian cows.

**Methods:**

In total, 38,792 first-parity cows were included. Pedigree inbreeding ($F_{PED}$) was computed and 75 k genotype data were used to compute genomic inbreeding, among others based on regions of homozygosity (ROH) in the genome ($F_{ROH}$).

**Results:**

Inbreeding depression was observed, e.g. a 1% increase in $F_{ROH}$ was associated with a 36.3 kg (SE = 2.4) decrease in 305-day milk yield, a 0.48 day (SE = 0.15) increase in calving interval and a 0.86 unit (SE = 0.28) increase in somatic cell score for day 150 through to 400. These effects equalled − 0.45, 0.12 and 0.05% of the trait means, respectively. When $F_{PED}$ was split into generation-based components, inbreeding on recent generations was more harmful than inbreeding on more distant generations for yield traits. When $F_{PED}$ was split into new and ancestral components, based on whether alleles were identical-by-descent for the first time or not, new inbreeding was more harmful than ancestral inbreeding, especially for yield traits. For example, a 1% increase in new inbreeding was associated with a 2.42 kg (SE = 0.41) decrease in 305-day fat yield, compared to a 0.03 kg (SE = 0.71) increase for ancestral inbreeding. There were no clear differences between effects of long ROH (recent inbreeding) and short ROH (ancient inbreeding).

**Conclusions:**

Inbreeding depression was observed for yield, fertility and udder health traits. For yield traits and based on pedigree, inbreeding on recent generations was more harmful than inbreeding on distant generations and there was evidence of purging. Across all traits, long and short ROH contributed to inbreeding depression. In future work, inbreeding depression and purging should be assessed in more detail at the genomic level, using higher density information and genomic time series.

## Background

Inbreeding depression is the decrease in mean performance due to mating between relatives. Many important traits in dairy cattle, such as yield and fertility traits, show inbreeding depression [1–4]. The genetic basis of inbreeding depression is increased homozygosity with inbreeding, which increases the frequency of unfavourable genotypes [5–7]. Although overdominance and epistasis may contribute to inbreeding depression, partial dominance is expected to account for the major proportion of inbreeding depression [6, 8, 9].

A variety of methods can be used to assess inbreeding depression. Traditionally, inbreeding depression has been assessed by regression of phenotypes on pedigree-based inbreeding coefficients [10–12]. Nowadays, with the wide availability of genotype data, pedigree-based inbreeding coefficients can be replaced by genomic inbreeding coefficients [1–3]. Genomic inbreeding can be computed from a genomic relationship matrix (GRM) or from the proportion of the genome covered by regions (or runs) of homozygosity (ROH) [13, 14]. Genomic inbreeding coefficients are expected to be more accurate than pedigree-based coefficients, because they account for Mendelian sampling variation (e.g. [15]) and do not depend on pedigree completeness and quality (e.g. [16]). Moreover, the use of ROH provides additional opportunities to distinguish recent from ancient inbreeding [1, 17, 18].

Not all inbreeding is expected to be equally harmful. Recent inbreeding (i.e. inbreeding arising from recent common ancestors) is expected to have a larger unfavourable effect than ancient inbreeding (i.e. inbreeding arising from more distant common ancestors). This hypothesis is based on the expected decrease in frequency of deleterious alleles over time, which is the result of (natural and/or artificial) selection. Since most deleterious alleles are (partially) recessive, inbreeding increases the efficiency of selection against these alleles by increasing homozygosity, which is called purging [9]. Purging is more likely to occur when there is strong selection pressure and when inbreeding accumulates slowly over many generations [9, 19].

With pedigree data, recent inbreeding may be distinguished from ancient inbreeding by including only a limited number of ancestral generations in the computation of inbreeding coefficients [18, 20]. Alternatively, one may use a purging-based approach to split the classical inbreeding coefficient into a new and an ancestral component, based on whether alleles are identical-by-descent (IBD) for the first time or have also been IBD in previous generations [21, 22]. The few studies that have applied the latter approach to commercial cattle populations found that the new inbreeding component was more harmful than the ancestral component, suggesting the presence of purging in these populations [4, 23].

With genomic data, age of inbreeding may be derived from the length of ROH [1, 17, 24]. Longer ROH reflect more recent inbreeding, because they have not yet been broken up by recombination. More specifically, the length of ROH derived from a common ancestor $G$ generations ago roughly follows an exponential distribution with a mean of $1/2G$ Morgan [24, 25]. Only a few studies have investigated the effect of ROH of different lengths on phenotypes in livestock, and the results of these studies vary [1, 18, 26].

The objective of this study was to evaluate the degree of inbreeding depression due to recent and ancient inbreeding in Dutch Holstein–Friesian dairy cattle. We expected to find stronger unfavourable effects for recent inbreeding compared to ancient inbreeding, because of selection against deleterious alleles over time (strengthened by purging). For a population of almost 40,000 genotyped cows, we determined the degree of inbreeding depression for yield, fertility and udder health traits. We used various pedigree-based and genomic inbreeding measures to compare these measures in terms of inbreeding depression. This study was performed in the context of artificial selection, meaning that all traits were under artificial selection and that natural selection will have had a relatively small contribution (or no contribution at all).

## Methods

### Animals and data

In total, 38,792 first-parity cows (fraction Holstein–Friesian > 87.5%, either red or black) from 233 herds were included. These cows calved in the period 2012–2016 and were from herds with a data-agreement with the Dutch-Flemish cattle improvement cooperative (CRV; Arnhem, the Netherlands). Initially, 47,254 first-parity cows from 440 herds during the 2012–2016 period were considered. From this initial dataset, herds with less than 10 genotyped cows per year were discarded ($n_{herds}$ = 207; $n_{cows}$ = 8462) in order to exclude herds in which only a few cows were occasionally genotyped.

Pedigree, genotype and phenotype data were provided by CRV. The total pedigree comprised 167,924 individuals. To assess pedigree completeness, the number of complete generations (NCG) and the complete generation equivalent (CGE) were computed. The CGE was computed as the sum of $$\left( {{\raise0.7ex\hbox{$1$} \!\mathord{\left/ {\vphantom {1 2}}\right.\kern-0pt} \!\lower0.7ex\hbox{$2$}}} \right)^{n}$$ of all known ancestors of an individual, with $$n$$ being the number of generations between the individual and a given ancestor. To limit the effect of missing pedigree information on results, cows with a NCG lower than 3 and/or a CGE lower than 10 were excluded from pedigree-based analyses (n = 1731). The mean NCG and CGE in the remaining cows equalled 6.5 generations and 12.5 generation-equivalents, respectively.

Cows were genotyped with the Illumina BovineSNP50 BeadChip (versions v1 and v2) or the CRV custom-made 60 k Illumina panel (versions v1 and v2). Genotypes were imputed to 76 k from the different panels, following Druet et al. [27]. Prior to imputation, single nucleotide polymorphisms (SNPs) with a call rate lower than 0.85, a minor allele frequency (MAF) lower than 0.025 or a difference of more than 0.15 between observed and expected heterozygosity were discarded. In addition, SNPs with an unknown position on the Btau4.0 genome assembly were discarded. The final dataset contained 75,538 autosomal SNPs.

Yield, fertility and udder health traits were considered. For yield, the 305-day milk yield (MY; in kg), 305-day fat yield (FY; in kg), and 305-day protein yield (PY; in kg) were included. For fertility, the calving interval (CI; in days), interval calving to first insemination (ICF; in days), interval first to last insemination (IFL; in days), and conception rate (CR; in %) were included. For udder health, the mean somatic cell scores for day 5 through to 150 (SCS150; in units) and day 151 through to 400 (SCS400; in units) were included. Somatic cell scores were calculated as 1000 + 100*[log2 of cells/mL].

### Inbreeding measures

Various inbreeding measures were used to assess inbreeding depression and distinguish recent from ancient inbreeding. These measures were divided into four groups: (1) pedigree generation-based measures, (2) pedigree purging-based measures, (3) ROH-based measures, and (4) GRM-based inbreeding.

#### Pedigree generation-based measures

The classical inbreeding coefficient based on all information in the pedigree ($F_{PED}$) was calculated with PEDIG [28]. The $F_{PED}$ was defined as the pedigree-based probability that two alleles at a random locus in an individual were IBD [29]. In addition to $F_{PED}$, inbreeding coefficients based on the first $n$ ancestral generations ($F_{PEDn}$), with $n$ ranging from 4 to 8, were computed with the *vanrad.f* program in PEDIG [28, 30]. Inbreeding for specific age classes was computed as the difference between successive coefficients (e.g. inbreeding on ancestors from 5 generations ago was computed as $F_{PED5} - F_{PED4}$; abbreviated as $F_{PED5 - 4}$). The $F_{PED8 - 7}$ was chosen as the most ancient category, because of the limited pedigree completeness for more ancient generations (e.g. only 78 cows had a NCG > 8) (see Additional file 1: Figure S1). The $F_{PED4}$ was chosen as the most recent category, because very few individuals were inbred on ancestors in the first ancestral generations (see Additional file 2: Figure S2).

#### Pedigree purging-based measures

Based on the hypothesis of purging, a few additional pedigree-based measures were calculated. Following Kalinowski et al. [21], the $F_{PED}$ was split into two components: an ancestral component ($F_{ANC}$) and a new component ($F_{NEW}$). The $F_{ANC}$ was defined as the probability that alleles were IBD while they had already been IBD in at least one ancestor, and $F_{NEW}$ was the probability that alleles were IBD for the first time in the pedigree of the individual. The ancestral history coefficient ($AHC$) introduced by Baumung et al. [22] was also calculated. $AHC$ was defined as the number of times that a random allele had been IBD during pedigree segregation [22]. Kalinowski’s inbreeding coefficients and the $AHC$ were obtained by gene dropping, using 10^6^ replications. The in-house script used for gene dropping is available upon request.

**Fig. 1:**
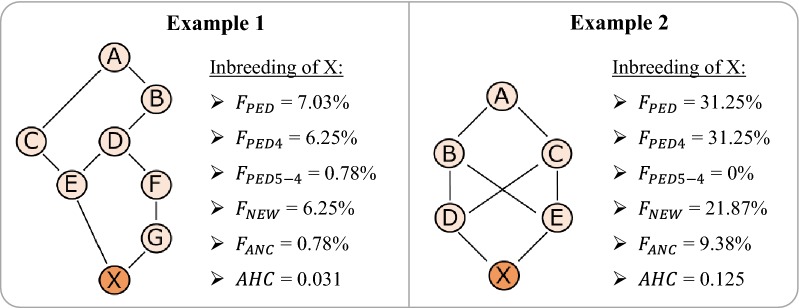
Example pedigrees illustrating differences between pedigree-based inbreeding measures for individual X. $F_{PED}$: classical pedigree inbreeding based on all available information; $F_{PED4}$: inbreeding based on first 4 generations; $F_{PED5 - 4}$: difference between inbreeding based on 5 and on 4 generations; $F_{NEW}$: Kalinowski’s new inbreeding, i.e. probability that alleles in X are IBD for the first time; $F_{ANC}$: Kalinowski’s ancestral inbreeding, i.e. probability that X is IBD for allele that has already been IBD in an ancestor; $AHC$: ancestral history coefficient, i.e. the number of times that a random allele from X has been IBD during pedigree segregation

To illustrate the differences between all pedigree-based inbreeding measures, two example pedigrees are provided (Fig. 1). In example (1), the $F_{PED}$ of individual X equals 7.03%, since it is the sum of the inbreeding on ancestor A (0.5^7^) and on ancestor D (0.5^4^). Since ancestor A is in the 5th ancestral generation and D is in the first 4 generations, $F_{PED5 - 4}$ equals the partial inbreeding on A (i.e. 0.5^7^) and $F_{PED4}$ equals the partial inbreeding on D (0.5^4^). $F_{ANC}$ is the probability that X is IBD for an allele that was already IBD in an ancestor, which in example (1) has to be ancestor E (since E is the only inbred ancestor). $F_{ANC}$ can be manually calculated by multiplying the probability that E is IBD for an allele of A (0.5^4^) with the probability that X inherits this allele from E given that E is IBD (1) and with the probability that X inherits this allele through D-F-G-X given that D is a carrier of the allele (0.5^3^). Thus, it is equal to 0.78% (i.e. 0.5^7^). In example (2), the $F_{PED}$ of individual X is higher (31.25%) than in example (1), while $F_{PED5 - 4}$ equals 0% based on the known information. The calculation of $F_{ANC}$ in example (2) depends on both D and E, since both ancestors are inbred. $F_{ANC}$ can be derived manually by tracing the possible genotype combinations. Individual A has two alleles, alleles 1 and 2. Consider the scenario in which individual B inherits allele 1 from A such that B has genotype 1/3, with 3 referring to a random allele inherited from the unknown parent of B. The possible genotypes of C are 1/4 and 2/4, where 4 is a random allele inherited from the unknown parent of C. If the genotype of C is 1/4, there are four possible genotypes for D and E (namely 1/1, 1/4, 3/1 and 3/4), resulting in 16 possible combinations of D and E and in 64 genotype possibilities for X. Among these 64 possibilities, there are 12 possibilities with X being 1/1 while D and/or E are 1/1 (four of which occur when D and E are both 1*/*1; the others occur when D or E is 1/1 while the other is 1/3 or 1/4). If C has genotype 2/4, while B is 1/3, there are also 64 genotype possibilities for X, but for none of these possibilities X will be IBD. Thus, if B is 1/3, there are 12 out of 128 possibilities for which X is IBD for allele 1 while D and/or E is also IBD for this allele. Similarly, if B is 2/3, there are 12 out of 128 possibilities for which X is IBD for allele 2 while D and/or E are also IBD for this allele. Therefore, the $F_{ANC}$ equals 24 out of 256 (i.e. 9.38%).

#### ROH-based measures

The scanning window approach implemented in the Plink 2.0 software [31] was used to identify ROH. The following criteria were set to define a ROH: (i) a minimum physical length of 1 Mb, (ii) a minimum of 10 SNPs, (iii) a minimum density of one SNP per 100 kb, (iv) a maximum of one heterozygous call within a ROH, and (v) a maximum gap of 500 kb between consecutive SNPs. A scanning window of 10 SNPs, with a maximum of one heterozygote per window, was used.

**Fig. 2:**
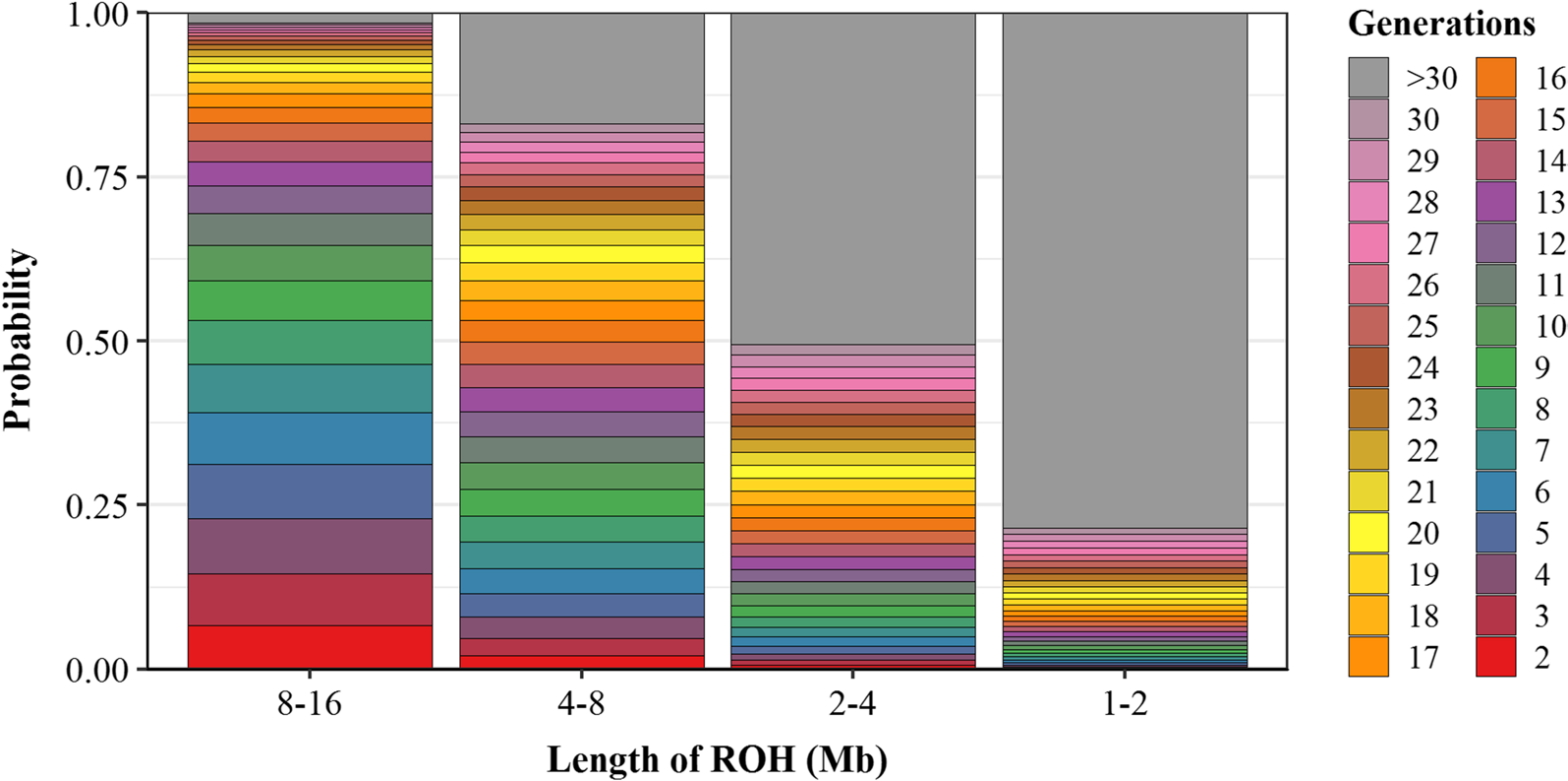
Expected age of inbreeding (in ancestral generations) for ROH classes, based on underlying exponential distributions. Note that this figure is an approximation, assuming a uniform distribution of inbreeding across ancestral generations, a uniform recombination rate across the genome and sexes, and a genetic distance of 1 Morgan per 100 Mb

After identification, ROH were classified into five length classes: (i) > 16 Mb, (ii) 8 to 16 Mb, (iii) 4 to 8 Mb, (iv) 2 to 4 Mb, and (v) 1 to 2 Mb. The expected age of inbreeding increased from the first to the last class, since shorter ROH reflect more ancient inbreeding. To illustrate this in more detail, the expected age was determined for each length category (Fig. 2). The expected age of inbreeding was based on the concept that the length of ROH derived from a common ancestor $G$ generations ago follows an exponential distribution with mean $1/2G$ Morgan [24, 25]. For simplicity, a mean genetic distance of 1 Morgan per 100 Mb [32] was used and it was assumed that recombination rates were uniform across the genome and across sexes. Note that non-uniform recombination rates may result in deviations from the exponential distribution. For example, Speed and Balding [24] performed extensive simulations for the human genome and found that length of ROH was best approximated with a gamma distribution with a shape parameter of 0.76. Since recombination rates may differ across the bovine genome and across sexes [32], Fig. 2 only provides a rough approximation of the expected length per ROH length class.

For each ROH length class, the inbreeding coefficient was calculated as the proportion of an individual’s autosome that was covered by ROH of that class (e.g. $F_{ROH > 16}$). Autosome length was corrected for uncovered regions (i.e. ends of chromosomes and gaps of more than 500 kb without SNPs) and the corrected autosome length was 2469 Mb. A total inbreeding coefficient based on all ROH ($F_{ROH}$) was also computed.

#### GRM-based inbreeding

Genomic inbreeding coefficients ($F_{GRM}$) were obtained as a measure of marker homozygosity. First, a genomic relationship matrix (GRM) was computed with *calc_grm* [33], according to the method of VanRaden [14]. Then, inbreeding coefficients were derived as the diagonal of the GRM minus 1 (since the relationship of an individual with itself equals 1 plus its inbreeding coefficient). When computing the GRM, allele frequencies were fixed to 0.5, such that $F_{GRM}$ was equivalent to the proportion of homozygous SNPs, except for a difference in scale [34].

### Statistical analyses

The degree of inbreeding depression was estimated by regressing phenotypes on inbreeding coefficients. For the total inbreeding measures ($F_{PED}$, $F_{ROH}$ and $F_{GRM}$), the following linear mixed model was used:

1 $$y_{ijk} = \mu + HY_{i} + month_{j} + \alpha *age_{k} + \beta *F_{k} + cow_{k} + e_{ijk} ,$$

where $HY_{i}$ is the $i{th}$ herd-year of calving (1165 classes), $month_{j}$ is the $j{th}$ month of calving (12 classes), $\alpha$ is the regression coefficient for $age_{k}$, which was the age at calving for the $k{th}$ cow, $\beta$ is the regression coefficient for $F_{k}$, which was the inbreeding coefficient for the $k{th}$ cow, $cow_{k}$ is the random genetic effect for the $k{th}$ cow, and $e_{ijk}$ is the random error term. The $cow$-effect was assumed to follow ${\text{N}}(0,{\mathbf{A}}\sigma_{a}^{2}$), where ${\mathbf{A}}$ is the numerator relationship matrix and $\sigma_{a}^{2}$ the additive genetic variance.

When $F_{PED}$ or $F_{ROH}$ was partitioned into classes based on inbreeding age, Model (1) was extended to fit these classes simultaneously (e.g. $F_{ROH > 16}$, $F_{ROH8 - 16}$, $F_{ROH4 - 8}$, $F_{ROH2 - 4}$ and $F_{ROH1 - 2}$):

2 $$y_{ijk} = \mu + HY_{i} + month_{j} + \alpha *age_{k} + \mathop \sum \limits_{l = 1}^{n} \beta_{l} *F_{kl} + cow_{k} + e_{ijk} ,$$

where $\beta_{l}$ is the regression coefficient for $F_{kl}$, which was the inbreeding coefficient for the $k{th}$ cow and the $l{th}$ inbreeding class, and $n$ is the number of inbreeding classes.

All analyses were performed with ASReml 4.1 [35]. Regression coefficients and corresponding standard errors (SE) for inbreeding measures were obtained from output. In addition, P-values for the Wald test were obtained from output and were used to check for significance of effects.

## Results

### Basic statistics for phenotypes and inbreeding measures

Descriptive statistics for the evaluated traits are in Table 1. Heritability estimates, obtained by running Model (1) without an inbreeding effect, were high for yield traits (0.36 to 0.47), moderate for somatic cell scores (0.11 and 0.14) and low for fertility traits (0.03 to 0.11).

**Table 1:** Number of cows (N), mean, standard deviation (SD), corrected phenotypic standard deviation (${\varvec{\upsigma}}_{{\mathbf{p}}}$), genetic standard deviation (${\varvec{\upsigma}}_{{\mathbf{a}}}$) and heritability (h^2^) for all evaluated traits

Trait	Trait unit	N	Mean	SD	${\varvec{\upsigma}}_{{\mathbf{p}}}$	${\varvec{\upsigma}}_{{\mathbf{a}}}$	h^2^ (SE)
MY	kg	38,778	8091	1375	1199	825	0.47 (0.02)
FY	kg	38,778	342	51.8	43.9	28.4	0.42 (0.02)
PY	kg	38,778	283	44.7	36.6	22.0	0.36 (0.02)
CI	days	34,864	394	67.2	65.3	18.5	0.08 (0.01)
ICF	days	34,937	77.6	30.0	27.2	7.9	0.08 (0.01)
IFL	days	34,937	39.9	56.1	55.4	12.3	0.05 (0.01)
CR	%	34,774	63.8	36.1	35.7	6.1	0.03 (0.01)
SCS150	1000 + 100*[log2 of cells/mL]	38,301	1568	138	134	45.5	0.11 (0.01)
SCS400	1000 + 100*[log2 of cells/mL]	37,068	1581	133	129	48.9	0.14 (0.01)

MY: 305-day milk yield; FY: 305-day fat yield; PY: 305-day protein yield; CI: calving interval; ICF: interval calving to first insemination; IFL: interval first to last insemination; CR: conception rate; SCS150: somatic cell score day 5 to 150; SCS400: somatic cell score day 151 to 400

**Fig. 3:**
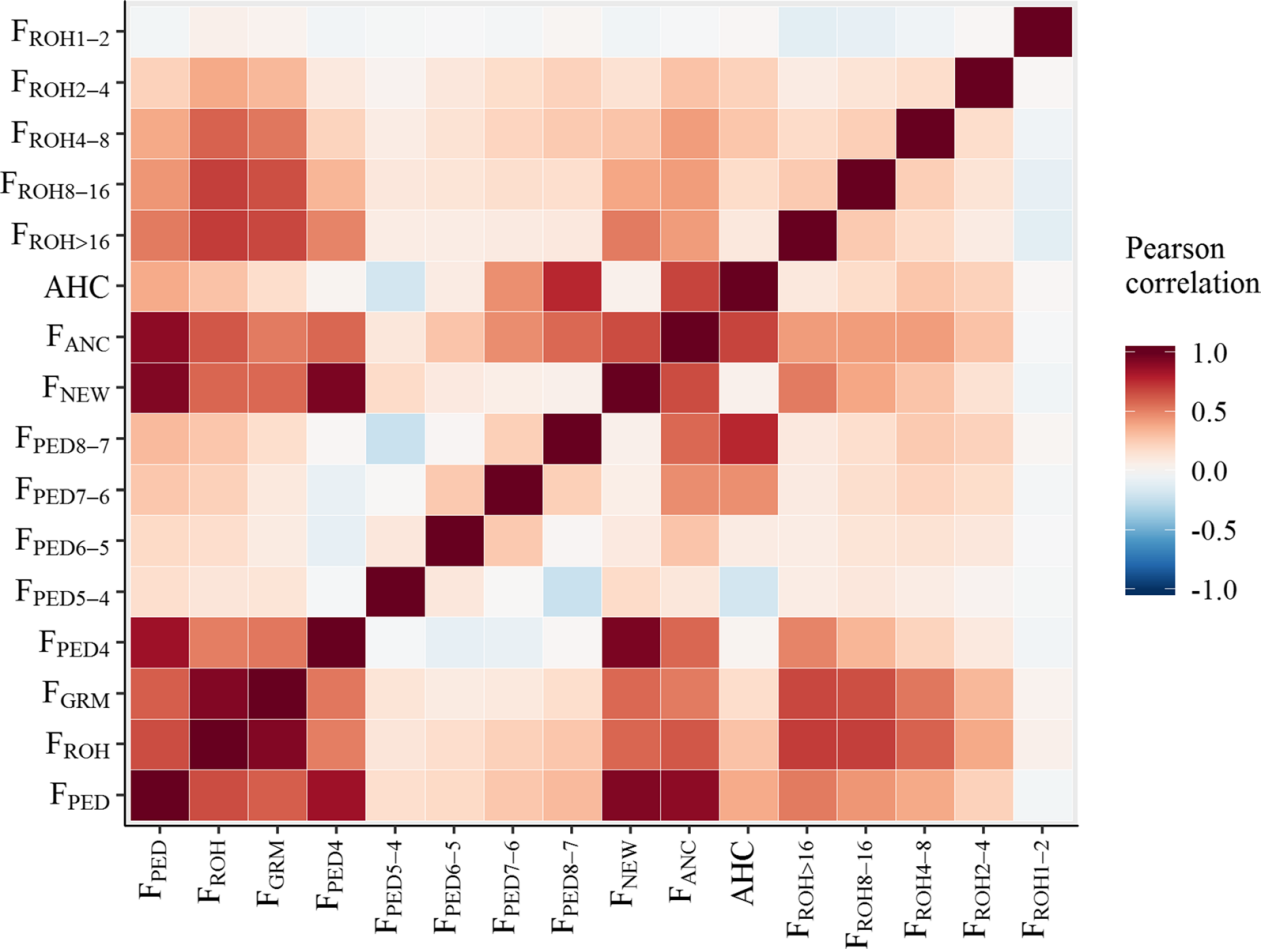
Heat map showing Pearson’s correlations between different inbreeding measures. $F_{PED}$: pedigree inbreeding based on all generations; $F_{ROH}$: inbreeding based on all regions of homozygosity; $F_{GRM}$: inbreeding based on genomic relationship matrix computed with allele frequencies of 0.5. $F_{PED4}$: pedigree inbreeding based on first 4 generations; $F_{PED5 - 4}$: difference between pedigree inbreeding based on 5 and on 4 generations; $F_{NEW}$: Kalinowski’s new inbreeding; $F_{ANC}$: Kalinowski’s ancestral inbreeding; $AHC$: ancestral history coefficient; $F_{ROH > 16}$: inbreeding based on regions of homozygosity longer than 16 Mb; $F_{ROH8 - 16}$: inbreeding based on regions of homozygosity of 8 to 16 Mb

Inbreeding based on ROH-coverage ($F_{ROH}$) was highly correlated with inbreeding based on marker homozygosity ($F_{GRM}$), with a Pearson correlation coefficient of 0.92 (Fig. 3). Pedigree-based inbreeding ($F_{PED}$) was moderately correlated with $F_{ROH}$ and $F_{GRM}$, with correlation coefficients of 0.66 and 0.61, respectively. The majority of cows (63%) were not inbred on ancestors in the first four ancestral generations, as illustrated by the distribution for $F_{PED4}$ (see Additional file 2: Figure S2). For cows that were inbred in the first four ancestral generations, clear peaks were visible at expected $F_{PED4}$-levels, for example at 0.78% (inbreeding on a single ancestor with an inbreeding loop of eight “steps”) and at 1.56% (a single loop of seven steps, or two loops of eight steps). In line with pedigree-based results, only a few cows had very long ROH (which indicate very recent inbreeding). About a fourth of the cows (26%) had no ROH > 16 Mb, 32% had a single ROH > 16 Mb, 21% had two ROH > 16 Mb and the remaining 21% had three or more ROH > 16 Mb. Pearson correlations suggest that the pedigree generation-based and the ROH-based measures partly captured the same age effects (Fig. 3). For example, $F_{PED4}$ showed a higher correlation with $F_{ROH > 16}$ ($r^{2}$ = 0.50) than with $F_{ROH8 - 16}$ (0.34), $F_{ROH4 - 8}$ (0.22), $F_{ROH2 - 4}$ (0.10) and $F_{ROH1 - 2}$ (− 0.03). Similarly, $F_{PED8 - 7}$ showed higher correlations with short ROH than with long ROH. Correlations among pedigree generation-based classes ranged from − 0.23 to 0.27 and correlations among ROH-classes ranged from − 0.10 to 0.26, suggesting rather independent inbreeding age classes. Notably, the $F_{ROH1 - 2}$ showed a negative or very low correlation (ranging from − 0.10 to 0.06) with all other calculated inbreeding measures, including overall homozygosity ($F_{GRM}$).

### Depression for total inbreeding measures

Inbreeding depression was observed for each of the total inbreeding measures ($F_{PED}$, $F_{ROH}$, $F_{GRM}$) and the estimated effects were significant for most traits (Table 2). For example, a 1% increase in $F_{ROH}$ was associated with a decrease in 305-day milk yield of 36.25 kg (*P* < 0.01), an increase in calving interval of 0.48 day (*P* < 0.01) and an increase in mean somatic cell score in day 151 to 400 of 0.80 units (*P* < 0.01). All estimated effects, including those that were not significant at the 0.05-level (e.g. for ICF), were unfavourable.

**Table 2:** Estimates of inbreeding depression for all traits and total inbreeding measures, expressed as the change in expected phenotype per 1% increase in inbreeding

Trait	$\varvec{F}_{{\varvec{PED}}}$		$\varvec{F}_{{\varvec{ROH}}}$		$\varvec{F}_{{\varvec{GRM}}}$	
	Estimate	SE	Estimate	SE	Estimate	SE
MY	− 37.95**	3.66	− 36.25**	2.35	− 48.07**	2.83
FY	− 1.54**	0.14	− 1.34**	0.09	− 1.60**	0.11
PY	− 1.27**	0.11	− 1.20**	0.07	− 1.55**	0.09
CI	0.46*	0.23	0.48**	0.15	0.62**	0.18
ICF	0.16	0.09	0.08	0.06	0.09	0.07
IFL	0.13	0.19	0.27*	0.12	0.42**	0.15
CR	− 0.31*	0.12	− 0.27**	0.08	− 0.36**	0.09
SCS150	0.58	0.44	0.30	0.28	0.44	0.34
SCS400	0.86*	0.43	0.86**	0.28	1.15**	0.33

MY: 305-day milk yield (kg); FY: 305-day fat yield (kg); PY: 305-day protein yield (kg); CI: calving interval (days); ICF: interval calving to first insemination (days); IFL: interval first to last insemination (days); CR: conception rate (%); SCS150 somatic cell score day 5 to 150 (1000 + 100*[log2 of cells/mL]); SCS400: somatic cell score day 151 to 400 (1000 + 100*[log2 of cells/mL])

$F_{PED}$: pedigree inbreeding based on all generations; $F_{ROH}$: inbreeding based on all regions of homozygosity; $F_{GRM}$: inbreeding based on genomic relationship matrix computed with allele frequencies of 0.5

Significance for non-nullity is indicated by stars (*P < 0.05; **P < 0.01)

To further illustrate differences in performance associated with changes in inbreeding, the expected phenotypes of cows with low (5% percentile) and high (95% percentile) inbreeding coefficients were compared (Table 3). These differences were computed only for traits that showed a significant depression effect for each of the total inbreeding measures. Differences between cows with low and high inbreeding coefficients were smaller for pedigree-based inbreeding than for genomic inbreeding measures. For example, the differences in 305-day milk yield between lowly and highly inbred cows were 198, 301 and 315 kg for $F_{PED}$, $F_{ROH}$ and $F_{GRM}$, respectively.

**Table 3:** Difference (Diff) between expected phenotypes of cows with low and high inbreeding, for significant traits and total inbreeding measures

Trait	$\varvec{F}_{{\varvec{PED}}}$			$\varvec{F}_{{\varvec{ROH}}}$			$\varvec{F}_{{\varvec{GRM}}}$		
	Low	High	Diff	Low	High	Diff	Low	High	Diff
MY	8175	7977	198	8227	7926	301	8232	7917	315
FY	345.4	337.4	8.0	347.0	335.9	11.1	346.7	336.2	10.5
PY	285.8	279.2	6.6	287.5	277.5	10.0	287.5	277.4	10.1
CI	393.0	395.4	− 2.4	392.2	396.2	− 4.0	392.2	396.2	− 4.0
IFL	39.6	40.3	− 0.7	38.9	41.1	− 2.2	38.7	41.4	− 2.7
CR	64.5	62.9	1.6	64.8	62.6	2.2	64.9	62.5	2.4
SCS400	1579	1583	− 4	1578	1585	− 7	1578	1585	− 7

MY: 305-day milk yield (kg); FY: 305-day fat yield (kg); PY: 305-day protein yield (kg); CI: calving interval (days); IFL: interval first to last insemination (days); CR: conception rate (%); SCS400: somatic cell score day 151 to 400 (1000 + 100*[log2 of cells/mL])

$F_{PED}$: pedigree inbreeding based on all generations; $F_{ROH}$: inbreeding based on all regions of homozygosity; $F_{GRM}$: inbreeding based on the genomic relationship matrix computed with allele frequencies of 0.5

Low and high inbreeding were defined as the 5% and 95% percentile, respectively. Low and high inbreeding equalled 2.8% and 8.0% for $F_{PED}$, 8.5% and 16.9% for $F_{ROH}$ and 25.9% and 32.4% for $F_{GRM}$

To compare depression effects across traits, the estimated regression coefficients from Table 2 were also expressed as the percentages of the corresponding trait means, as well as in phenotypic and genetic standard deviations (see Additional file 3: Table S1). When expressed in percentages of trait means, yield traits showed a relatively large depression effect (of 0.39 to 0.47%) and somatic cell scores a relatively small effect (of 0.02 to 0.05%). The effect for fertility differed across traits and inbreeding measures. It was relatively high for CR and IFL (0.33 to 0.67%) and intermediate for CI and ICF (0.11 to 0.21%). When compared in phenotypic standard deviations, yield traits showed the highest degree of inbreeding depression. When compared in genetic standard deviations, yield traits also showed the highest degree of inbreeding depression, in spite of the lower heritability of fertility and udder health traits. Only conception rate, which had a very low heritability of 0.03, showed a depression effect similar to that of yield traits when compared in genetic standard deviations.

### Depression for pedigree generation-based inbreeding components

**Fig. 4:**
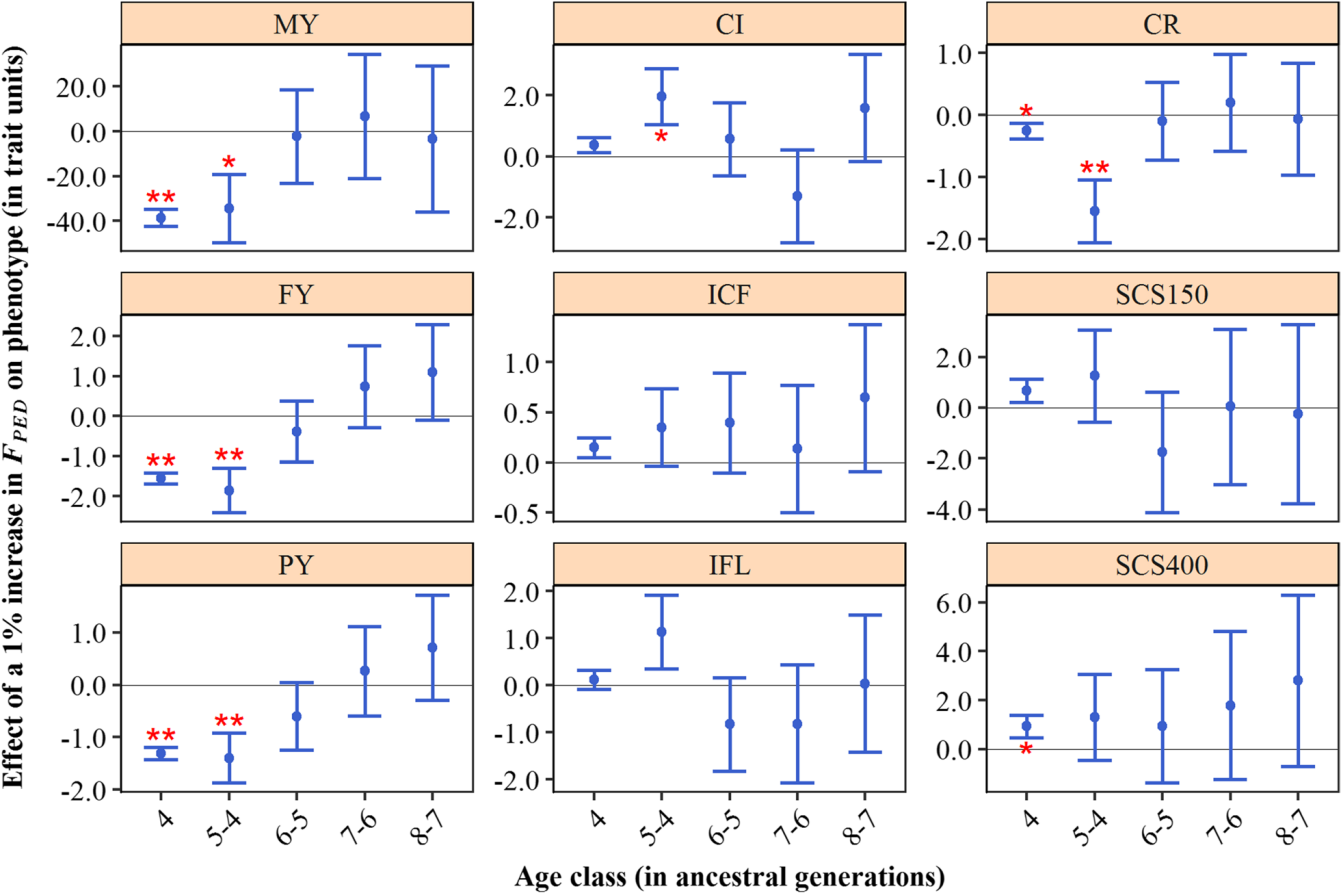
Effect of a 1% increase in pedigree inbreeding ($F_{PED}$) on phenotypes, for different age classes. Error bars represent one standard error and stars indicate significance for non-nullity (*P < 0.05; **P < 0.01). MY: 305-day milk yield (kg); FY: 305-day fat yield (kg); PY: 305-day protein yield (kg); CI: calving interval (days); ICF: interval calving to first insemination (days); IFL: interval first to last insemination (days); CR: conception rate (%); SCS150 somatic cell score day 5 to 150 (1000 + 100*[log2 of cells/mL]); SCS400: somatic cell score day 151 to 400 (1000 + 100*[log2 of cells/mL])

When $F_{PED}$ was split into generation-based classes, recent inbreeding significantly reduced milk, fat and protein yield whereas more ancient inbreeding had a non-significant neutral or even favourable effect (Fig. 4). For example, the estimated effects for 305-day protein yield from the most recent to the most ancient class were equal to − 1.3 kg (for $F_{PED4}$), − 1.4 kg ($F_{PED5 - 4}$), − 0.6 kg ($F_{PED6 - 5}$), 0.3 kg ($F_{PED7 - 6}$) and 0.7 kg ($F_{PED8 - 7}$). For fertility and udder health traits, estimated effects were generally not significantly different from zero and no clear pattern was visible for these traits. For example, the interval between calving and first insemination seemed to be unfavourably affected by all classes, but none of the effects was significant. For all traits, standard errors increased with age of inbreeding. This may be explained by a lower degree of variation for more ancient inbreeding (see Additional file 2: Figure S2).

### Depression for pedigree purging-based inbreeding components

**Fig. 5:**
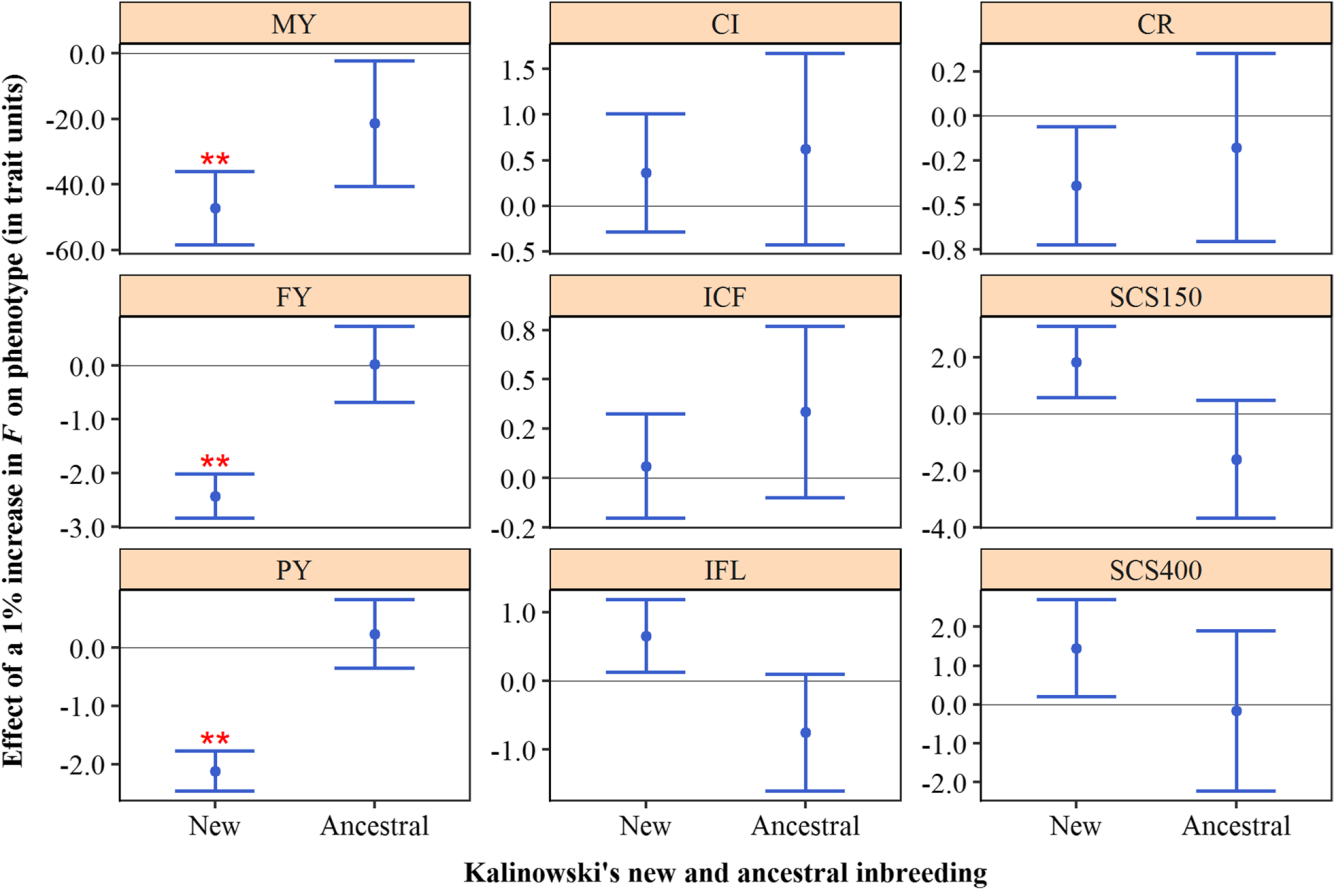
Effect of a 1% increase in Kalinowski’s new and ancestral inbreeding on phenotypes. Error bars represent one standard error and stars indicate significance for non-nullity (*P < 0.05; **P < 0.01). MY: 305-day milk yield (kg); FY: 305-day fat yield (kg); PY: 305-day protein yield (kg); CI: calving interval (days); ICF: interval calving to first insemination (days); IFL: interval first to last insemination (days); CR: conception rate (%); SCS150 somatic cell score day 5 to 150 (1000 + 100*[log2 of cells/mL]); SCS400: somatic cell score day 151 to 400 (1000 + 100*[log2 of cells/mL])

When $F_{PED}$ was split into Kalinowski’s new ($F_{NEW}$) and ancestral ($F_{ANC}$) components, new inbreeding significantly reduced milk, fat and protein yield, whereas ancestral inbreeding did not (Fig. 5). For example, a 1% increase in $F_{NEW}$ was associated with a 2.42 kg (SE = 0.41) decrease in 305-day fat yield, while a 1% increase in $F_{ANC}$ was associated with a 0.03 kg (SE = 0.71) increase in fat yield. For fertility and udder health traits, both new and ancestral inbreeding effects were not significantly different from zero. For most traits (MY, FY, PY, IFL, CR, SCS150, SCS400), the estimated effect of new inbreeding was more unfavourable than the effect of ancestral inbreeding. For some traits (e.g. IFL), the estimated effect of ancestral inbreeding was even slightly favourable, whereas the effect of new inbreeding was always unfavourable.

The $AHC$ had no significant effect on traits, except for a favourable effect on 305-day protein yield (Table 4). When $AHC$ was fitted simultaneously with $F_{PED}$, fat yield also tended to increase with an increase in $AHC$ (*P *<0.1). Interactions between $AHC$ and $F_{PED}$ were not significant.

**Table 4:** Effect of an increase in the ancestral history coefficient ($\varvec{AHC}$) on all traits, when a model with only the $\varvec{AHC}$ or with the $\varvec{AHC}$ and pedigree-based inbreeding ($\varvec{F}_{{\varvec{PED}}}$) was used

Trait	Model with only $\varvec{AHC}$		Model with $\varvec{AHC}$ and $\varvec{F}_{{\varvec{PED}}}$			
	$\varvec{AHC}$		$\varvec{AHC}$		$\varvec{F}_{{\varvec{PED}}}$	
	Estimate	SE	Estimate	SE	Estimate	SE
MY	157.1	306.5	403.7	307.0	− 38.3**	3.7
FY	9.4	11.1	20.2	11.1	− 1.6**	0.14
PY	24.5**	9.2	34.1**	9.2	− 1.31**	0.11
CI	11.5	14.7	7.0	14.9	0.44	0.23
ICF	6.8	6.1	5.3	6.2	0.15	0.09
IFL	− 11.2	11.8	− 12.9	12.0	0.17	0.19
CR	3.5	7.2	7.0	7.4	− 0.34**	0.12
SCS150	− 25.3	29.9	− 31.4	30.2	0.64	0.44
SCS400	− 3.2	30.0	− 11.4	30.3	0.88*	0.43

MY: 305-day milk yield (kg); FY: 305-day fat yield (kg); PY: 305-day protein yield (kg); CI: calving interval (days); ICF: interval calving to first insemination (days); IFL: interval first to last insemination (days); CR: conception rate (%); SCS150 somatic cell score day 5 to 150 (1000 + 100*[log2 of cells/mL]); SCS400: somatic cell score day 151 to 400 (1000 + 100*[log2 of cells/mL])

Significance for non-nullity is indicated by stars (*P < 0.05; **P < 0.01)

### Depression for ROH length-based inbreeding components

**Fig. 6:**
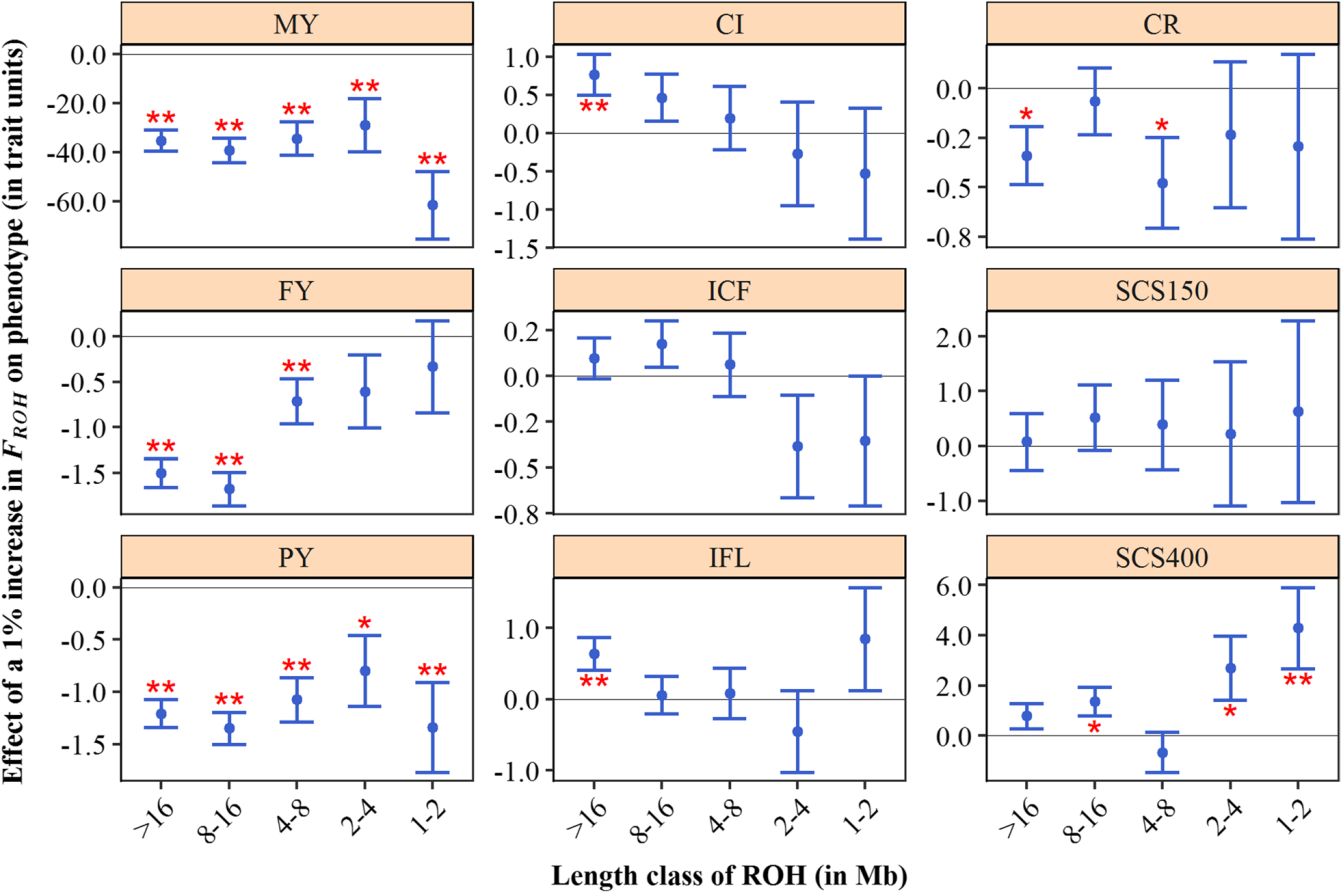
Effect of a 1% increase in ROH-based inbreeding ($F_{{ROH}}$) on phenotypes, for different ROH lengths. Error bars represent one standard error and stars indicate significance for non-nullity (*P < 0.05; **P < 0.01). MY: 305-day milk yield (kg); FY: 305-day fat yield (kg); PY: 305-day protein yield (kg); CI: calving interval (days); ICF: interval calving to first insemination (days); IFL: interval first to last insemination (days); CR: conception rate (%); SCS150 somatic cell score day 5 to 150 (1000 + 100*[log2 of cells/mL]); SCS400: somatic cell score day 151 to 400 (1000 + 100*[log2 of cells/mL])

When $F_{ROH}$ was split into classes based on ROH length (> 16, 8–16, 4–8, 2–4 and 1–2 Mb), the effect of these classes differed across traits (Fig. 6). For 305-day milk yield, for example, all five classes showed a significant decrease in yield per 1% increase in inbreeding, with a slightly stronger effect for ancient inbreeding ($F_{ROH1 - 2}$; effect of − 60 kg) than for more recent inbreeding (longer ROH-classes; effects varying from − 29 to − 40 kg). For 305-day fat yield, an increase in $F_{ROH > 16}$ and $F_{ROH8 - 16}$ was associated with a decrease in yield, while for shorter ROH this decrease was less pronounced. For fertility and udder health traits, most effects were not significantly different from zero. However, some of these traits did show a trend. For calving interval and for the interval between calving and first insemination, inbreeding based on long ROH seemed to increase these intervals, whereas that based on shorter ROH seemed to decrease these intervals. In contrast, for somatic cell score for day 151 through to 400, there seemed to be a larger unfavourable effect of short ROH compared to long ROH. Across all traits, standard errors were larger for inbreeding based on short ROH compared to long ROH. This may be the result of less variation in inbreeding based on short ROH (see Additional file 2: Figure S2).

## Discussion

### Inbreeding depression and its costs

Estimates of pedigree-based inbreeding depression were comparable to those reported in previous studies. For example, a 1% increase in pedigree inbreeding has previously been associated with a reduction in 305-day milk yield of 20 to 30 kg [12, 36, 37] and with an increase in calving interval of 0.2 to 0.7 days [10, 36, 37]. Inbreeding depression for somatic cell score has also been observed before [37–39], but estimates were not directly comparable because of different scales and because of the use of separate measures for early (SCS150) and late (SCS400) lactation in the current study. In general, the accuracy of pedigree-based results depends largely on pedigree quality and completeness. Incomplete pedigrees may lead to downward bias of inbreeding coefficients and, therefore, to misleading estimates of inbreeding depression [40]. In an attempt to limit this bias, we decided to include only the individuals with a NCG of at least three generations and a CGE of at least 10 equivalents in this study.

Estimates of inbreeding depression based on genomic inbreeding measures were similar to those estimated for pedigree-based inbreeding and to those reported in other studies. In US Holstein–Friesian cattle, Bjelland et al. [2] found a decrease in 205-day milk yield of 20 and 47 kg for a 1% increase in $F_{ROH}$ and $F_{GRM}$, respectively. They also observed an increase in days open (a trait similar to calving interval) of 1.72 and 1.06 days for $F_{ROH}$ and $F_{GRM}$, respectively. They did not observe an effect on SCS. In Australian Holstein–Friesian cattle, Pryce et al. [1] estimated inbreeding depression based on a $F_{GRM}$ measure that was corrected for allele frequencies of the contemporary population. They found that a 1% increase in their $F_{GRM}$-estimates was associated with a decrease in lactation yields for milk, fat and protein of 28 kg, 1.3 kg and 0.9 kg, respectively. In addition, they observed a slight increase in calving interval of 0.12 days, although this increase was not significant. As illustrated by the current and previous studies, genomic measures of inbreeding can be effectively used to estimate the effects of inbreeding on performance. In fact, we found that $F_{ROH}$ and $F_{GRM}$ captured more phenotypic differences between lowly and highly inbred cows than $F_{PED}$ (Table 3), in spite of the larger estimated change in phenotype per 1% increase in $F_{PED}$ compared to $F_{ROH}$ (Table 2). This finding was in line with the results of Bjelland et al. [2] and is the direct result of a wider distribution for $F_{ROH}$ compared to $F_{PED}$ (see Additional file 2: Figure S2). The finding that $F_{PED}$ captures less inbreeding depression than $F_{ROH}$ and $F_{GRM}$ may be explained by the random nature of recombination and segregation, which is captured with genomic measures but not with pedigree. Since there will be more measurement errors in pedigree inbreeding than in genomic inbreeding, there will be more attenuation or “flattening” of the slope towards zero for $F_{PED}$ (a statistical phenomenon known as regression dilution). For the various inbreeding measures, which Keller et al. [17] investigated in their simulation study, ROH-based inbreeding showed the highest correlation with the homozygous mutation load. Our results suggest that $F_{GRM}$ and $F_{ROH}$ capture similar effects of inbreeding depression at the population level, which is not surprising because of the high correlation between these two measures ($r^{2}$ = 0.93 in this study).

Costs of inbreeding should be considered in the framework of a breeding program. For example, for a trait such as 305-day milk yield, we estimated a reduction of around 38 kg per 1% increase in pedigree-based inbreeding. If we consider that the pedigree-based inbreeding level in Dutch Holstein–Friesian cattle has increased from around 0.5% in 1980 to around 4.5% in 2010 [41–43], this would roughly imply a mean loss of 150 kg due to inbreeding depression. Such a loss is small compared to the realised genetic progress in the same period, which was equal to approximately 2200 kg [44]. Although the rate of inbreeding has increased with the introduction of genomic selection [41], contrary to expectation [45], the increased genetic gains [46] are expected to still outweigh the losses caused by inbreeding depression. It should be noted that overall costs will be larger than the cost for single traits, especially since components of economic return may combine multiplicatively rather than additively [47]. In addition, it is important to realise that inbreeding will also affect traits that were not included in the present study, such as stillbirths [23]. Previous economic analyses of inbreeding depression suggested lifetime losses per cow in the order of tens of US dollars per 1% increase in inbreeding [10, 12, 39]. These analyses confirm that, by affecting various traits, inbreeding depression reduces net income. Combined with the importance of conserving genetic diversity for future adaptability, the costs of inbreeding depression provide incentive to monitor and manage inbreeding in dairy cattle populations.

### Recent inbreeding is more harmful than ancient inbreeding and evidence of purging

The main objective of this study was to evaluate the hypothesis that recent inbreeding is more harmful than ancient inbreeding. This hypothesis was based on the expected decrease in frequency of deleterious alleles over time as a result of selection, strengthened by purging. Computer simulations have shown that purging is more effective when selection pressure is strong and when inbreeding accumulates slowly over many generations [9, 19]. We expected that purging would have occurred in the Dutch Holstein–Friesian population, because the population has undergone decades of intense artificial selection and inbreeding has accumulated (at least until 2012) at approximately 0.13% per year [41–43].

Pedigree-based results support our hypothesis. For yield traits, inbreeding on recent generations was more harmful than inbreeding on more distant generations (Fig. 4). In addition, there was evidence of purging for these traits (Fig. 5). For most traits, Kalinowski’s $F_{NEW}$ was more harmful than Kalinowski’s $F_{ANC}$ (Fig. 5). For some traits, the estimated effect of $F_{ANC}$ was even favourable. In other words, to be IBD for alleles that were already IBD in the past had a neutral or favourable effect, whereas to be IBD for alleles for the first time was generally unfavourable. These findings are in line with the hypothesis of purging, which states that loci that have undergone inbreeding in the past have been exposed to an increased selection efficiency (against deleterious recessive alleles), compared to loci that have not undergone inbreeding before. Our results are largely in line with previous studies that have investigated purging in commercial cattle populations [4, 23]. In German Holstein–Friesian cattle, Hinrichs et al. [23] studied the effects of new and ancestral inbreeding on reproductive traits. They found that a 1% increase in $F_{NEW}$ was associated with a decrease in birthweight of 11.9 kg, while a 1% increase in $F_{ANC}$ was associated with an increase in birthweight of 41.6 kg. They also observed a significant increase in the rate of stillbirths for $F_{NEW}$, while $F_{ANC}$ showed a slight reduction in stillbirths that was not significant. In Irish Holstein–Friesian cattle, Mc Parland et al. [4] investigated the effects of new and ancestral inbreeding on yield and fertility traits. They found that a 1% increase in $F_{NEW}$ was associated with a decrease in 305-day milk, fat and protein yields of 32.4 kg, 2.4 kg and 1.1 kg, respectively. They also found unfavourable effects for $F_{ANC}$, but these effects were less strong, namely 8.9 kg, 0.5 kg and 0.3 kg, respectively. For calving interval, they estimated an increase of 4.1 and 0.6 days for $F_{NEW}$ and $F_{ANC}$, respectively. Differences across studies may be partly explained by the way that $F_{NEW}$ and $F_{ANC}$ have been fitted. In this study and in the study of Hinrichs et al. [23], the $F_{NEW}$ and $F_{ANC}$ were fitted simultaneously in the model, thereby accounting for the correlation between the two measures ($r^{2}$ = 0.67 in this study). In the study of Mc Parland et al. [4], however, $F_{NEW}$ and $F_{ANC}$ were fitted individually.

Differences between effects of recent and ancient inbreeding (Fig. 4) and between effects of Kalinowski’s $F_{NEW}$ and $F_{ANC}$ (Fig. 5) were most apparent for yield traits, which is in accordance with Mc Parland et al. [4]. This finding may be explained by the selection history of Dutch Holstein–Friesian cattle. Targeted selection for fertility and udder health has taken place only since these traits were included in the breeding goal around the year 2000, whereas selection for yield traits has taken place for many more decades [42]. Therefore, there has been less time for selection to act on alleles that affect fertility and udder health traits compared to alleles that affect yield traits.

In addition to Kalinowski’s new and ancestral inbreeding, we also considered the ancestral history coefficient ($AHC$). $AHC$ is defined as the number of times that a random allele in an individual has been IBD in the individual’s pedigree [22]. The rationale behind this recently introduced measure is that purging is not fully efficient and that the probability of purging increases with the number of times the alleles have been IBD. In other words, an allele that has been IBD many times in an individual’s pedigree is more likely to have a neutral or positive effect on traits under selection, compared to an allele that has been IBD only once or never before. An increase in $AHC$, therefore, is expected to be associated with a favourable effect on the phenotype. Indeed, we observed a few favourable effects, i.e. an increase in protein yield and a tendency for an increase in fat yield (Table 4). Most traits showed no significant effect, but the estimate was generally favourable. In Thoroughbred horses, Todd et al. [48] found a strong positive association between $AHC$ and racing performance. Compared to their study, where the mean $AHC$ was 1.97 (SD = 0.09), the mean $AHC$ in the current study was rather low at 0.31 (SD = 0.05). This can be explained by the very comprehensive pedigree of the Thoroughbred population, which dates back to the late eighteenth century, with individuals from 2000 to 2010 having a mean CGE of 24.6 [48].

A purging-based measure that we did not include in this study is Ballou’s [49] ancestral inbreeding coefficient ($F_{ANC\_BAL}$). The $F_{ANC\_BAL}$ is defined as the probability that any allele in an individual has been IBD in an ancestor at least once [49]. It can be calculated by using an iterative formula [49] or with gene dropping [50], where gene dropping provides more robust estimates by accounting for dependence between $F_{ANC\_BAL}$ and $F_{PED}$ [50]. To assess the effect of purging, one has to include the product of $F_{ANC\_BAL}$ and $F_{PED}$ in the model [4, 49, 51], because $F_{ANC\_BAL}$ does not consider the IBD-probability for an individual itself. The product of $F_{ANC\_BAL}$ and $F_{PED}$ is the probability that an individual is IBD for an allele that was already IBD in at least one ancestor, which is in fact the definition of Kalinowski’s $F_{ANC}$ [21]. Similarly, the product of ($1 - F_{ANC\_BAL}$) and $F_{PED}$ is equivalent to the $F_{NEW}$ of Kalinowski. Because of this equivalence, we decided to include only Kalinowski’s measures in this study.

More recently, an inbreeding-purging (IP) model was proposed to assess purging based on genealogical information [52]. This model, which was developed in a conservation biology context, predicts how fitness evolves in a population undergoing inbreeding by means of a purged inbreeding coefficient ($g$). $g$ is the traditional inbreeding coefficient weighted by the reduction in frequency of deleterious alleles induced by purging. Using simulations, López-Cortegano et al. [53] showed that inbreeding depression estimates based on the IP model are similar to those obtained using Ballou’s approach, with smaller standard errors for the IP model. We considered using the IP model for the current study. Since the model and associated software (PURGd) have been developed outside the context of artificially selected populations, various limitations exist for its application to livestock data. First, random effects cannot be fitted in the model, making it impossible to directly correct for additive genetic relationships. To overcome this limitation, one could first run an animal model in a different software environment (e.g. ASReml) and subsequently use the residuals as phenotypes for the IP model. This two-step process is not desirable, because it will affect the inbreeding depression estimates. Second, the model assumes that inbreeding load is due to deleterious alleles that have a low initial frequency in the (base) population. In the context of livestock breeding, where animals are selected based on a breeding goal composed of various traits [42], we do not expect that alleles that are deleterious for a single trait necessarily segregate at a low frequency. Given these limitations, we decided not to use the IP model in the current study. For future research, it would be valuable to explore further the application of the IP model in (livestock) populations undergoing artificial selection.

### Long and short ROH contribute to inbreeding depression

We expected that inbreeding based on long ROH (recent inbreeding) would be associated with stronger depression effects than inbreeding based on short ROH (ancient inbreeding). For some traits (e.g. fat yield and calving interval) our results were in line with this hypothesis, but for other traits there was no clear pattern across ROH-length classes or there was even a pattern in the opposite direction (Fig. 6). Overall, both long and short ROH seemed to contribute to inbreeding depression.

Only a few studies have investigated the effect of ROH of different lengths on phenotypes in livestock populations, with various results [1, 18, 26]. In Austrian Fleckvieh, Ferenčaković et al. [18] found stronger inbreeding depression for number of spermatozoa when considering both long and short ROH (e.g. > 2 Mb) than when considering only long ROH (e.g. > 16 Mb). For autosome 3 in Iberian pigs, Saura et al. [26] observed that inbreeding based on long ROH (> 5 Mb) significantly decreased the number of piglets born, whereas inbreeding based on short ROH (0.5 to 5 Mb) had a non-significant favourable effect. In Australian Holstein–Friesian cattle, Pryce et al. [1] observed a stronger depression effect for 305-day milk yield when only very long ROH were included than when also shorter ROH were included. To further investigate and compare our results to the findings of Pryce et al. [1], we also ran Model (1) for cumulative ROH-based inbreeding coefficients (i.e. $F_{ROH > 16}$, $F_{ROH > 8}$, $F_{ROH > 4}$, $F_{ROH > 4}$ and $F_{ROH > 1}$). We obtained a similar trend (see Additional file 4: Figure S3) as Pryce et al. [1], with $F_{ROH > 16}$ showing the strongest effect and the inclusion of shorter ROHs reducing the effect size. The difference between results for fitting multiple length classes simultaneously (Fig. 6) and for fitting cumulative measures one by one (see Additional file 4: Figure S3) may be due to the correlations between classes. We believe that fitting length classes simultaneously provides the most accurate estimates, since this approach accounts for the correlations between classes.

Based on computer simulations, Keller et al. [17] concluded that long ROH correlate better with the homozygous mutation load than short ROH for a population with an effective population size of 100 (which is the approximate size of the Holstein–Friesian population [41–43]). Functional predictions of deleterious variation have led to inconsistent conclusions as to whether short or long ROH harbour more deleterious genetic variants [54, 55]. For the human genome, Szpiech et al. [54] predicted that long ROH (of several Mb) are enriched with deleterious variants compared to short ROH. In contrast, for four Danish cattle breeds Zhang et al. [55] predicted that short (< 0.1 Mb) and medium (0.1 to 3 Mb) ROH are significantly enriched in deleterious variants compared to long (> 3 Mb) ROH. For domestic dogs, Sams and Boyko [56] recently reported that the relative risk of a ROH carrying a known deleterious variant is similar across ROH of different lengths, suggesting that ROH of all lengths may contribute to inbreeding depression in dogs. This latter finding is more in line with our results, where both short and long ROH seem to contribute to inbreeding depression.

There are various aspects that affect the accuracy of identification of ROH and the inference of inbreeding age based on ROH. First, the density of the SNP panel determines the size of ROH that can be accurately identified. Previous studies have shown that the use of a 50 k panel may result in false positive ROH shorter than 5 Mb and especially in many false positives ROH shorter than 2 Mb [57, 58]. For a more accurate estimation of ancient inbreeding, and to apply this approach to even more generations in the past, high-density SNP data or sequence data is required. Second, in this study we assumed a uniform recombination rate, while it actually varies across the genome (e.g. [32]). A ROH of a given physical length in a region with high recombination will reflect more ancient inbreeding than a ROH of the same length in a region with low recombination. One may account for this effect by computing ROH based on genetic distances. However, this is rarely done in practice, since it requires a high-quality linkage map [59]. Third, recent inbreeding may mask more ancient inbreeding [26]. If both chromosomes at a position in the genome trace back to a distant common ancestor, you expect to find a short ROH. If the same region also traces back to a recent common ancestor, then you would observe only the long ROH. As a result, one may expect a negative correlation between recent and ancient ROH-based inbreeding. In Iberian pigs, Saura et al. [26] report such a negative correlation of -0.641 between inbreeding based on short ROH (0.5 to 5 Mb) and based on long ROH (> 5 Mb). In this study, we found some negative correlations between the very short ROH ($F_{ROH1 - 2}$) and the other classes (Fig. 3). However, these negative correlations could also be an artefact of the unreliable estimation of short ROH. To correct for the masking of ancient inbreeding by recent inbreeding, one could subtract the length of long ROH of the total length of the genome when calculating $F_{ROH}$ for short ROH. The effect of this or other correction(s) should be investigated in future studies. Lastly, various approaches can be used to identify ROH. In this study, we applied the sliding window approach implemented in Plink 2.0 [31], with a set of (rather arbitrary) rules to define a ROH. As an alternative to this rule-based approach, one may use a Hidden Markov model (HMM) to identify ROH and infer age of inbreeding [59, 60]. In the future, it would be valuable to compare the different approaches and investigate the benefit of using linkage maps to infer inbreeding age based on ROH.

As sequencing costs continue to decrease, genomic data (including that of cows) will become increasingly available. This offers opportunities to perform large-scale analyses on genomic inbreeding depression based on high-density information, e.g. to identify regions associated with inbreeding depression [1, 18, 61]. In addition, genomic time series (consisting of genomic data of an individual and its ancestors) could be used to study purging in more detail at the genomic level.

## Conclusions

Inbreeding depression was observed for yield, fertility and udder health traits in Dutch Holstein–Friesian dairy cattle. Observed inbreeding depression was stronger for yield traits than for fertility and udder health traits, when compared in (phenotypic or genetic) standard deviations. Genomic inbreeding captured more inbreeding depression than pedigree-based inbreeding at the population level. For yield traits and based on pedigree information, inbreeding on recent generations was found to be more harmful than inbreeding on distant generations and there was evidence of purging. Based on ROH, there was no clear difference between the effects of long ROH (recent inbreeding) and short ROH (ancient inbreeding). Future work should investigate inbreeding depression and purging in more detail at the genomic level, using higher density information and genomic time series.

## Supplementary information

**Additional file 1: Figure S1.** Distribution of the number of complete generations (NCG) and complete generation equivalent (CGE) for cows included in pedigree-based analyses (n = 37,061).

**Additional file 2: Figure S2.** Distributions of inbreeding measures and the AHC (n = 37,061 for pedigree-based measures and n = 38,792 for genomic measures). The mean ($\overline{x}$) and standard deviation ($SD$) are also shown. $F_{PED}$: pedigree inbreeding based on all generations; $F_{ROH}$: inbreeding based on all regions of homozygosity; $F_{GRM}$: inbreeding based on genomic relationship matrix computed with allele frequencies of 0.5; $F_{PED4}$: pedigree inbreeding based on first 4 generations; $F_{PED5 - 4}$: difference between pedigree inbreeding based on 5 and on 4 generations; $F_{NEW}$: Kalinowski’s new inbreeding; $F_{ANC}$: Kalinowski’s ancestral inbreeding; $AHC$: ancestral history coefficient; $F_{ROH > 16}$: inbreeding based on regions of homozygosity longer than 16 Mb; $F_{ROH8 - 16}$: inbreeding based on regions of homozygosity of 8 to 16 Mb.

**Additional file 3: Table S1.** Estimates of inbreeding depression for all traits^1^ and total inbreeding measures^2^, expressed in percentage of trait means (% of $\overline{x}$), in corrected phenotypic standard deviations ($\sigma_{p}$) and in genetic standard deviations ($\sigma_{a}$). The results for $\sigma_{p}$ and $\sigma_{a}$ were multiplied by 100. Estimates correspond to the estimates in Table 2.

**Additional file 4: Figure S3.** Effect of a 1% increase in ROH-based inbreeding ($F_{ROH}$) for cumulative measures. Error bars represent one standard error and stars indicate significance for non-nullity (*P < 0.05; **P < 0.01). MY: 305-day milk yield (kg); FY: 305-day fat yield (kg); PY: 305-day protein yield (kg); CI: calving interval (days); ICF: interval calving to first insemination (days); IFL: interval first to last insemination (days); CR: conception rate (%); SCS150 somatic cell score day 5 to 150 (1000 + 100*[log2 of cells/mL]); SCS400: somatic cell score day 151 to 400 (1000 + 100*[log2 of cells/mL]).

## Data Availability

All information supporting the results is included in the text, figures and tables of this article. The dataset is not publicly available due to commercial restrictions.
