# Supplementary figures and images for: Inbreeding depression due to recent and ancient inbreeding in Dutch Holstein–Friesian dairy cattle
Source: Genet Sel Evol. 2019 Sep 27;51:54. doi: 10.1186/s12711-019-0497-z (PMC6764141; doi:10.1186/s12711-019-0497-z)

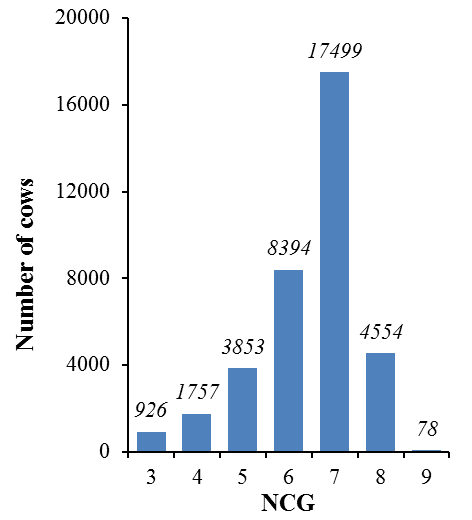

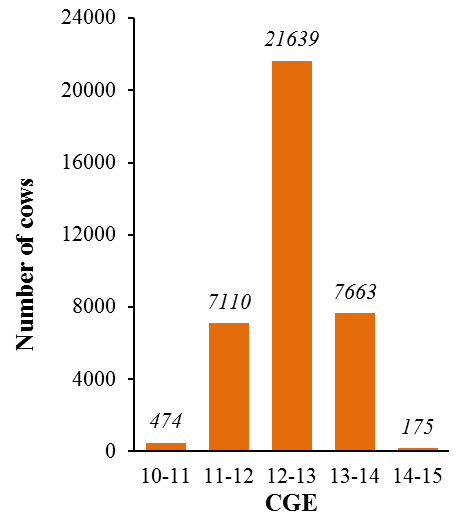

Supplement: Supplementary file 1 — Additional file 1: Figure S1. Distribution of the number of complete generations (NCG) and complete generation equivalent (CGE) for cows included in pedigree-based analyses (n = 37,061). [file 12711_2019_497_MOESM1_ESM.docx]

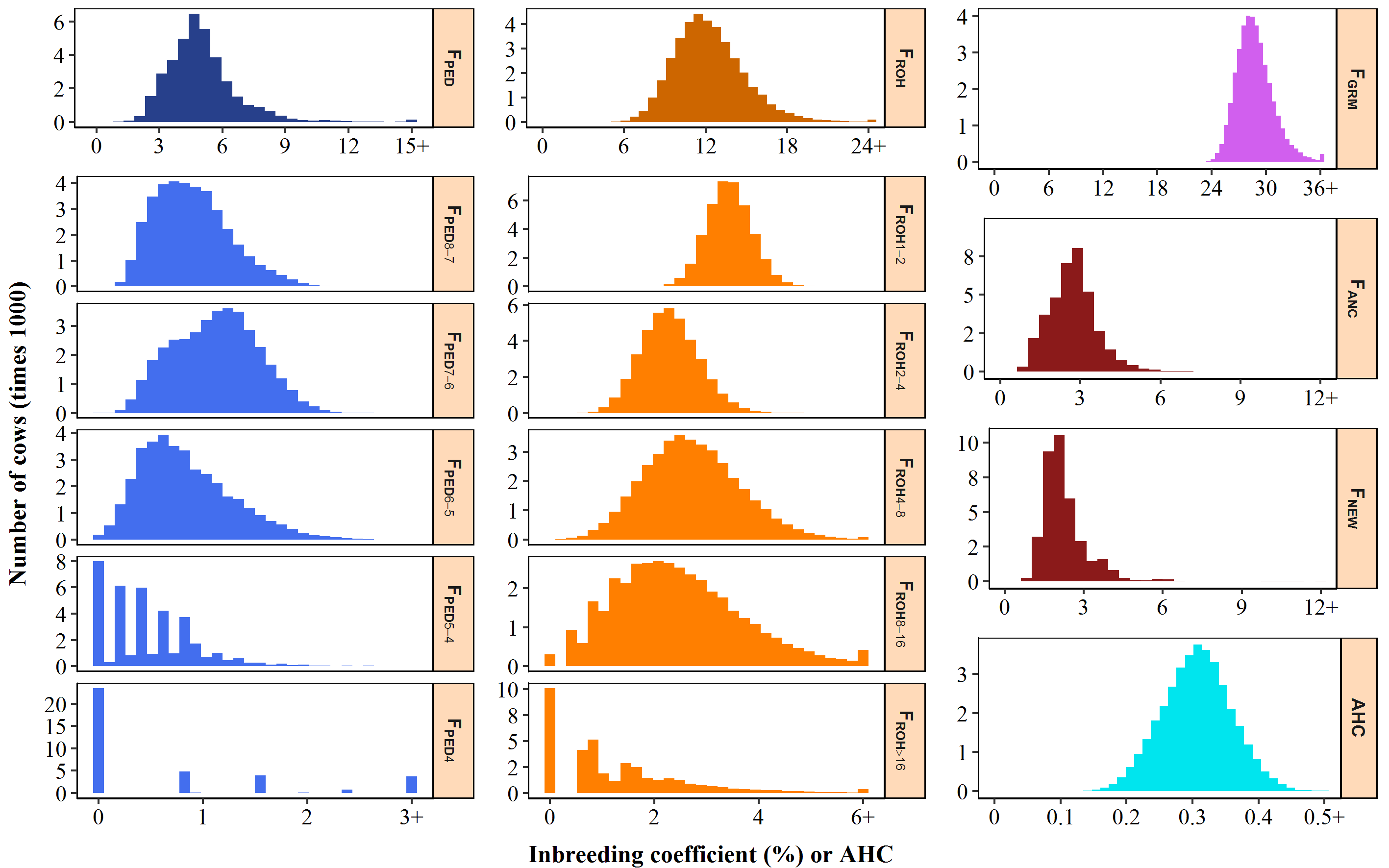


*= 0.84
= 0.44*

*= 5.03
= 1.81*

*= 0.49
= 0.45*

*= 0.90
= 0.36*

*= 1.12
= 0.40*

*= 0.76
= 1.59*

*= 2.73
= 0.92*

*= 12.29
= 2.66*

*= 2.48
= 1.25*

*= 3.43
= 0.43*

*= 2.30
= 0.55*

*= 1.35
= 1.40*

*= 2.29
= 1.08*

*= 0.31
= 0.05*

*= 2.73
= 0.91*

*= 28.79
= 2.10*

Supplement: Supplementary file 2 — Additional file 2: Figure S2. Distributions of inbreeding measures and the AHC (n = 37,061 for pedigree-based measures and n = 38,792 for genomic measures). The mean ($\overline{x}$x¯) and standard deviation ($SD$SD) are also shown. $F_{PED}$FPED: pedigree inbreeding based on all generations; $F_{ROH}$FROH: inbreeding based on all regions of homozygosity; $F_{GRM}$FGRM: inbreeding based on genomic relationship matrix computed with allele frequencies of 0.5; $F_{PED4}$FPED4: pedigree inbreeding based on first 4 generations; \docume [file 12711_2019_497_MOESM2_ESM.docx]

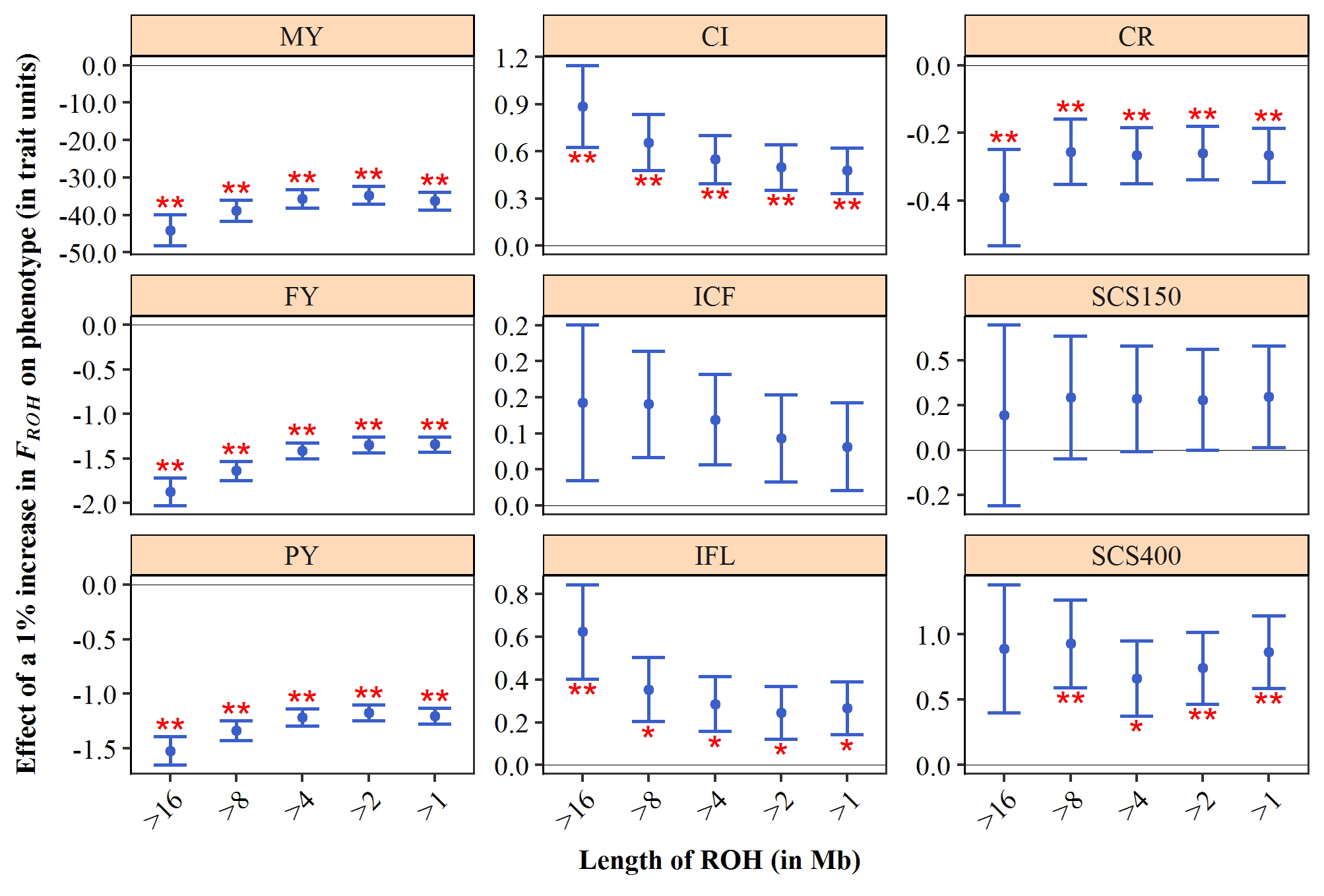

Supplement: Supplementary file 4 — Additional file 4: Figure S3. Effect of a 1% increase in ROH-based inbreeding ($F_{ROH}$FROH) for cumulative measures. Error bars represent one standard error and stars indicate significance for non-nullity (*P < 0.05; **P < 0.01). MY: 305-day milk yield (kg); FY: 305-day fat yield (kg); PY: 305-day protein yield (kg); CI: calving interval (days); ICF: interval calving to first insemination (days); IFL: interval first to last insemination (days); CR: conception rate (%); SCS150 somatic cell score day 5 to 150 (1000 + 100*[log2 of cells/mL]); SCS400: somatic cell score day 151 to 400 (1000 + 100*[log2 of cells/mL]). [file 12711_2019_497_MOESM4_ESM.docx]
