# Supplementary material for: Inbreeding depression due to recent and ancient inbreeding in Dutch Holstein–Friesian dairy cattle
Source: Genet Sel Evol. 2019 Sep 27;51:54. doi: 10.1186/s12711-019-0497-z (PMC6764141; doi:10.1186/s12711-019-0497-z)
Supplement: Supplementary file 3 — Additional file 3: Table S1. Estimates of inbreeding depression for all traits1 and total inbreeding measures2, expressed in percentage of trait means (% of $\overline{x}$x¯), in corrected phenotypic standard deviations ($\sigma_{p}$σp) and in genetic standard deviations ($\sigma_{a}$σa). The results for $\sigma_{p}$σp and $\sigma_{a}$σa were multiplied by 100. Estimates correspond to the estimates in Table 2. [file 12711_2019_497_MOESM3_ESM.docx]

|  | $F_{PED}$ | | |  | $F_{ROH}$ | | |  | $F_{GRM}$ | | |
| --- | --- | --- | --- | --- | --- | --- | --- | --- | --- | --- | --- |
| Trait | in % of $\bar{x}$ | in $\sigma_{p}$ | in $\sigma_{a}$ |  | in % of $\bar{x}$ | in $\sigma_{p}$ | in $\sigma_{a}$ |  | in % of $\bar{x}$ | in $\sigma_{p}$ | in $\sigma_{a}$ |
| MY | -0.47 | -3.16 | -4.60 |  | -0.45 | -3.02 | -4.39 |  | -0.45 | -4.01 | -5.83 |
| FY | -0.45 | -3.51 | -5.42 |  | -0.39 | -3.05 | -4.72 |  | -0.39 | -3.64 | -5.63 |
| PY | -0.45 | -3.46 | -5.76 |  | -0.42 | -3.28 | -5.46 |  | -0.42 | -4.24 | -7.05 |
| CI | 0.12 | 0.70 | 2.47 |  | 0.12 | 0.73 | 2.57 |  | 0.12 | 0.95 | 3.35 |
| ICF | 0.21 | 0.60 | 2.07 |  | 0.11 | 0.30 | 1.04 |  | 0.11 | 0.34 | 1.17 |
| IFL | 0.33 | 0.24 | 1.08 |  | 0.67 | 0.48 | 2.16 |  | 0.67 | 0.76 | 3.42 |
| CR | -0.49 | -0.87 | -5.08 |  | -0.42 | -0.74 | -4.33 |  | -0.42 | -1.02 | -5.93 |
| SCS150 | 0.05 | 0.43 | 1.27 |  | 0.02 | 0.22 | 0.65 |  | 0.02 | 0.33 | 0.96 |
| SCS400 | 0.05 | 0.67 | 1.76 |  | 0.05 | 0.67 | 1.77 |  | 0.05 | 0.89 | 2.35 |

^1^MY: 305-day milk yield (kg); FY: 305-day fat yield (kg); PY: 305-day protein yield (kg); CI: calving interval (days); ICF: interval calving to first insemination (days); IFL: interval first to last insemination (days); CR: conception rate (%); SCS150 somatic cell score day 5 to 150 (1000+100*[log2 of cells/mL]); SCS400: somatic cell score day 151 to 400 (1000+100*[log2 of cells/mL]).

^2^$F_{PED}$: pedigree inbreeding based on all generations; $F_{ROH}$: inbreeding based on all regions of homozygosity; $F_{GRM}$: inbreeding based on genomic relationship matrix computed with allele frequencies of 0.5.
